# Risk assessment model for sleep disturbance based on gastrointestinal myoelectrical activity in middle-aged and elderly people

**DOI:** 10.3389/fpsyt.2023.1183108

**Published:** 2023-06-23

**Authors:** Shuming Ji, Baichuan Li, Chenxing Zhu, Guohui Jiang, Yusha Tang, Lei Chen

**Affiliations:** ^1^Department of Clinical Research Management, West China Hospital, Sichuan University, Chengdu, China; ^2^Department of Neurology, Joint Research Institution of Altitude Health, West China Hospital, Sichuan University, Chengdu, China; ^3^Department of Neurology, Affiliated Hospital of North Sichuan Medical College, Nanchong, Sichuan, China

**Keywords:** sleep disturbance, Electrogastroenterography, LASSO regression, decision curve analysis, ROC curve

## Abstract

**Background:**

Sleep disturbance has become a considerable factor affecting the quality of life for middle-aged and elderly people; however, there are still many obstacles to screening sleep disturbance for those people. Given the growing awareness of the association between gastrointestinal function and sleep disturbance, our study aims to predict the risk of sleep disturbance using gastrointestinal electrophysiological signals.

**Methods:**

The Pittsburgh Sleep Quality Index and gastrointestinal electrophysiological signals of 914 participants in western China were used to establish the model. Demographic characteristics and routine blood test were collected as covariates. Participants were randomly assigned into two sets with a 7:3 ratio for training and validation. In the training set, the least absolute shrinkage and selection operator (LASSO) regression and stepwise logistic regression were used, respectively for variables selection and optimization. To assess the model performance, receiver operator characteristic (ROC) curve, calibration curve and decision curve analysis (DCA) were utilized. Then, validation was performed.

**Results:**

Thirteen predictors were chosen from 46 variables by LASSO regression. Then, age, gender, percentage of normal slow wave and electrical spreading rate on the pre-meal gastric channel, dominant power ratio on the post-meal gastric channel, coupling percent and dominant frequency on the post-meal intestinal channel were the seven predictors reserved by logistic regression. The area under ROC curve was 0.65 in the training set and 0.63 in the validation set, both exhibited moderate predictive ability. Furthermore, by overlapping the DCA results of two data-sets, there might be clinical net benefit if 0.35 was used as reference threshold for high risk of sleep disturbance.

**Conclusion:**

The model performs a worthy predictive potency for sleep disturbance, which not only provides clinical evidence for the association of gastrointestinal function with sleep disturbance, but also can be considered as an auxiliary assessment for screening sleep disturbance.

## Introduction

Sleep disturbance is one of the most prevalent mental disorders, especially among the middle-aged and elderly people (1), which generally manifest insomnia, narcolepsy, poor sleep quality, and obstructive sleep apnea (OSA) syndrome (2). According to epidemiological surveys, the prevalence of sleep disturbance increases significantly with aging and affects more than half of those over the age of 60 (3); and moreover, the COVID-19 pandemic has exacerbated the effects of sleep disturbance (4). Sleep disturbance could drastically reduces a person’s quality of life (5), and raises the chance of developing a mental disorder, such as depression or anxiety, or both, especially in the middle-aged and elderly individuals (6–9). Furthermore, it has been shown that elderly individuals who have sleep disturbance exhibit a higher risk of developing neurological diseases including Parkinson’s and Alzheimer’s diseases as well as stroke, migraine, and cognitive impairment (10–14). On the other hand, elderly people with sleep disturbance are more reliant on sleeping pills (15, 16), which adds to their illness burden, impacts the ability to communicate with others, and injures the energy in social activities during the day (17).

A general community screening is required for such individuals due to the significance of sleep disturbance in the senior population, although there are some challenges. Currently, patient-rated questionnaires, such the Insomnia Severity Index (ISI) and Pittsburgh Sleep Quality Index (PSQI), are trustworthy tools for estimating the severity of sleep disturbance (18). However, since the use of PSQI and ISI necessitates that participants have the necessary cognitive capacities and educational levels, it is challenging to become effective and widely accessible community screening tools for all middle-aged and elderly people or those with cognitive decline. In addition, polysomnography could provide precise estimations of sleep duration and quality, but due to its costly and time-consuming drawbacks, it is also difficult to become a routine clinical procedure (19). Therefore, a practical, effective, and objective instrument is urgently needed to aid to the screening of sleep disturbances in the middle-aged and elderly people.

In recent years, the bidirectional regulatory mechanism of gut-brain axis has attracted a lot of scientific attention, which highlights the close connection between the gastrointestinal homeostasis and the health of the central nervous system (CNS), and reveals the significant effect of gastrointestinal function changes on the CNS (20). The conception of the gut-brain axis also corresponds to an old Chinese proverb saying that a disturbed stomach makes it impossible to go asleep, which was an early exploration in the association between gastrointestinal function and sleep disturbance.

Numerous studies have shown this close association between gastrointestinal dysfunction and sleep disturbance. For instance, some clinical studies have found that gastrointestinal diseases like inflammatory bowel disease, gastro-oesophageal reflux disease, digestive disease, and functional gastrointestinal disorders are more likely develop symptoms of sleep disturbance, and these diseases that most frequently impair sleep are acid-related (21–23). Additionally, Zhe Wang et al. (24) pioneered the microbiota-gut-brain axis that the gut microbiota is bidirectional correlated with sleep behavior, which may contribute to the regulation of sleep quality. These findings raise the prospect of predicting the risk of sleep disturbance by using diagnostic tools for gastrointestinal function.

Electrogastroenterogram (EGEG) is a non-invasive method that uses cutaneous electrodes applied to the abdominal skin across the stomach and intestine to capture myoelectrical activity (25). The myoelectric activity of the gastrointestinal tract is primarily composed of slow wave and spinal potential. Since its popularization in the 1990s, EGEG has been used as an auxiliary method of diagnosing a variety of gastrointestinal functional disorders (26). Furthermore, W C Orr et al. (27) revealed that patients with irritable bowel syndrome had significantly different manifestations of EGEG during sleep compared to normal people. Anjiao Peng et al. (28) also demonstrated that patients with rapid eye movement sleep behavior disorder also had irregular changes of EGEG. Based on these lines of evidences, our study aims to compare the differences in gastrointestinal myoelectrical activity by EGEG between patients with sleep disturbance and healthy controls, and establishes a risk assessment model with the expectation of considering as an auxiliary assessment for sleep disturbance in the middle-aged and elderly people.

## Methods and materials

### Subjects

In this cross-sectional research, participants over the age of 40 were recruited from 60 communities in western China between January 2020 and December 2021. All subjects voluntarily participated in our study and signed informed consent, and they were required to possess the necessary knowledge and communication ability to complete the relevant questionnaire and clinical diagnostic. The exclusion criteria for subjects included: 1) Subjects diagnosed with gastrointestinal diseases such as gastritis and gastric ulcer, within the last 6 months, 2) Subjects with gastrointestinal discomfort such as diarrhea and constipation, 3) Subjects with a history of drug use within the past week, 4) Subjects with severe cardio, liver and kidney dysfunction or metabolic diseases such as diabetes, 5) Subjects with major mental illness. Following that, all subjects were told to abstain from alcohol and follow a light diet for 3 days in order to complete the EGEG test, along with a sleep disturbance questionnaire and a routine blood test on the same day. All clinical examinations and questionnaire assessments were conducted jointly by neurology physicians from the West China hospital and community health service staff. For each subject, general information on age, gender, body mass index (BMI), and the history of smoking and alcohol drinking was collected, clinical information including the results of PSQI questionnaire, routine blood tests, and EGEG examination was also collected. This study was approved by the Ethics Committee of West China Hospital of Sichuan University (No. 2018–491, 2022–1,138).

### Routine blood test

We required each participant in our study undergo a routine blood test, and collected their most common blood glucose and lipid information such as glucose, triglyceride (TG), total cholesterol (Tch), high density lipoprotein (HDL) and low density lipoprotein (LDL).

### EGEG records

**Figure 1:**
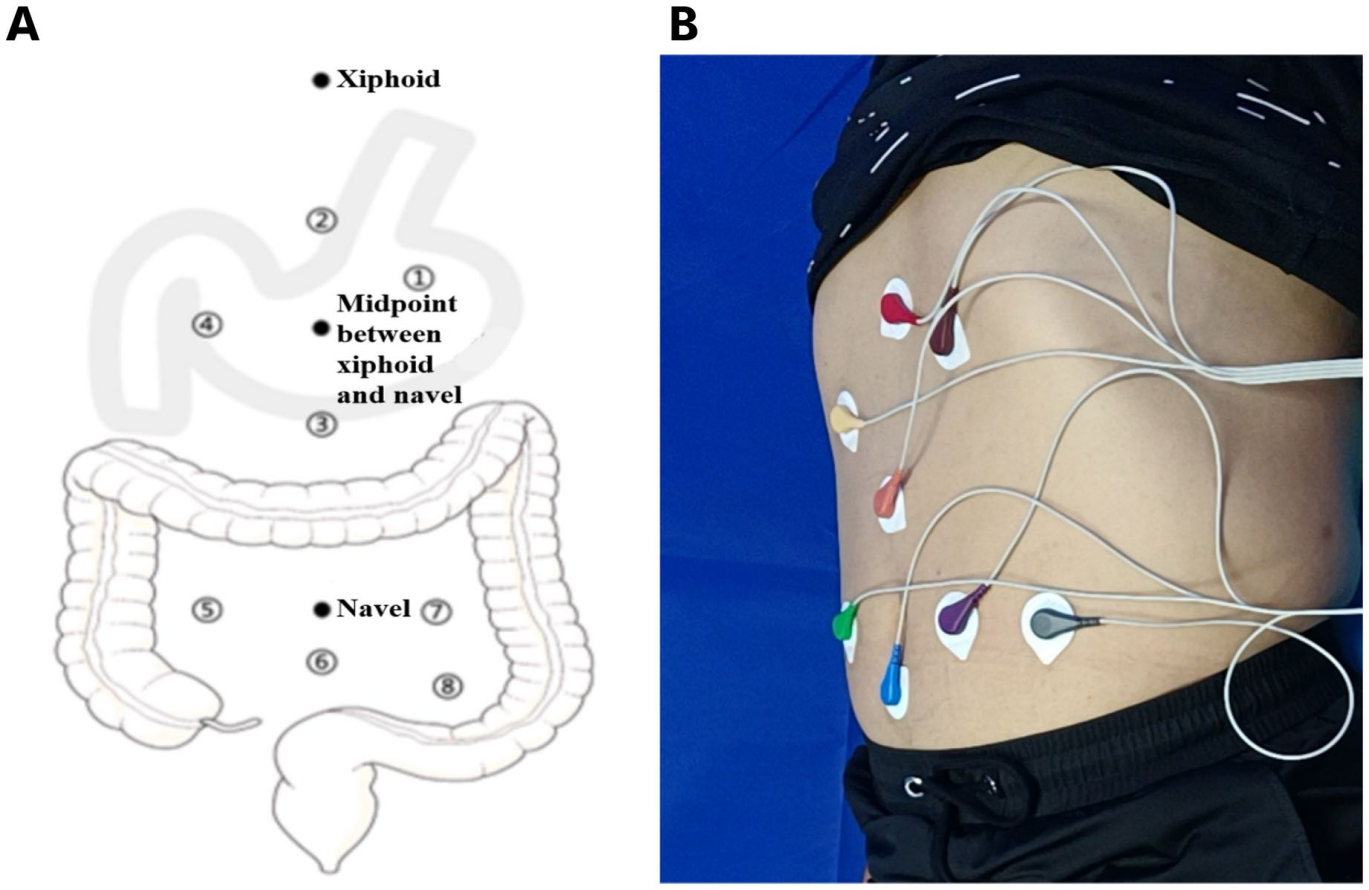
**(A)** A pattern diagram of electrodes positioning for EGEG recording. Eight electrodes from gastric and intestinal channels reflect the myoelectrical activity, including four gastric electrodes (corpus gastricum, gastric lesser curvature, gastric greater curvature, gastric antrum), and four intestinal electrodes (ascending colon, transverse colon, descending colon, rectum). **(B)** A sample diagram of the positioning process in EGEG examination. EGEG, electrogastroenterogram.

We used an eight-channel gastrointestinal electromyograph (XDJ-S8, Hefei Kaili Company, Hefei, China) to measure the gastrointestinal myoelectrical activity. Prior to the EGEG examination, all participants were instructed to fast for at least 6 hours and to refrain from consuming alcohol and spicy, greasy, or irritating foods for at least 3 days. In a supine position, the measurement of EGEG was performed. Eight gastrointestinal electrodes, including four gastric electrodes (corpus gastricum, gastric antrum, gastric lesser curvature, and gastric greater curvature), and four intestinal electrodes (ascending colon, transverse colon, descending colon, and rectum), were positioned on the abdominal skin (Hanjie Company. Ltd., Shanghai, China), as shown in Figure 1.

The reference electrode was positioned on the medial wrist of the right hand, and the grounding electrode was positioned on the medial ankle of the right leg. Participants were instructed to remain still for the whole 6 minutes of the pre-meal EGEG recording. Following that, they were received a mealtime functional load test, which providing about 200 kcal of food, and accepted a 6 minutes post-meal EGEG recording after waiting 5 minutes.

### EGEG data processing

The background noise was filtered out from the EGEG recordings by setting a high cutoff frequency of 0.1 Hz and a low cutoff frequency of 0.008 Hz. The EGEG recordings were produced at sampling rate of 1 Hz.

After visual inspection for artifacts, the raw EGEG data was automatically calculated by the computer alongside the EGEG, and the following indicators of each electrode were collected separately: 1) mean amplitude of waveform (MAW), 2) mean frequency of waveform (MFW), 3) electrical rhythm disturbance (ERD), 4) reaction area of waveform (RAW), 5) electrical spreading rate (ESR), 6) dominant frequency (DF), 7) dominant power ratio (DPR), 8) percentage of normal slow wave (PNSW), 9) coupling percent (CP).

Given the high degree of internal consistency among the electrode recording data on the gastric and intestinal channels, we merged the electrode indicators by averaging four gastric and four intestinal electrodes separately. Meanwhile, due to the EGEG tests were conducted pre-meal and post-meal, a total of 36 EGEG parameters were eventually derived for each participant.

### Sleep disturbance assessment

The PSQI questionnaire was used to assess sleep quality of the participants in the past month, and to diagnose whether they had a sleep disturbance. The PSQI questionnaire consists of 19 self-reported questions and five questions that should be answered by roommates, which are used only for clinical information and not tabulated in the scoring. The 19 self-reported questions were used to calculate the PSQI score, which could divided into seven components, with each component scored from 0 to 3. The seven components separately are: 1) subjective sleep quality, 2) sleep latency, 3) sleep duration, 4) habitual sleep efficiency, 5) sleep disturbances, 6) use of sleeping medication, and 7) daytime dysfunction. The sum of these components produces a global score, which ranges from 0 to 21, where a higher score indicates worse sleep quality. A total score over than eight points is considered as having a sleep disturbance, and whether the subjects had a sleep disturbance is the outcome variable in our study (29).

## Statistic analysis

### Variables selection

In linear regression models, the least absolute shrinkage and selection operator (LASSO) regression is a shrinkage and variable selection approach that might aid to the identification of significant predictors. LASSO regression could shrink a portion of the regression coefficients towards zero, and any predictors with a coefficient of zero are eliminated from the model. For forecasting the response variable, the remaining predictors with nonzero coefficients are regarded as being the most crucial (30). Using the type measure of-2log-likelihood and the binomial family to centralize and normalize the included variables, and the optimum lambda value was chosen by LASSO regression with 10 fold cross-validation. The best performing model was created using the “Lambda.min” setting.

According to the theoretical requirements for external validation, the total number of participants in our study were randomly split into a training set and a validation set in a 7:3 ratio. We included a total of 46 variables for variables screening, including the demographic characteristics, the results of routine blood test, and 36 EGEG parameters.

Considering the large number of variables included in this study, in order to ensure sufficient statistical efficiency of the assessment model in the training set, we required that the sample size of the training set should be at least 10 times more than the number of variables, and therefore the estimated total sample size for this study should be at least 658 people.

### Setting risk assessment model

The statistic analysis in our study consisted of two parts: variables selection and assessment of predictive power. The data in the training set was analyzed using the LASSO regression in order to select the optimal predictors from all the variables. The predictors chosen from LASSO regression was further optimized by using stepwise multi-variable logistic regression. Then, final version of risk assessment model was established and plotted the nomogram of the model. The odds ratio (OR) and 95% confidence interval (CI) were used in our study to define the contributions and to produce the nomogram.

Furthermore, several validation methods were used to evaluate the predicting efficiency of the risk assessment model, both into the training set and the validation set. The area under the receiver operating characteristic (ROC) curve was used to evaluate the performance of the risk assessment model in identifying true positive patients with sleep disturbance from participants. The calibration curve, accompanied by the Hosmer-Lemeshow test, was used to assess the calibration of this model (31). The decision curve analysis (DCA) was used to determine the clinical practicability of model according to searching the best net benefit under the different threshold probabilities (32). All analyzes were performed using R version 4.1.3 with packages glmnet and rms, and the significance level was set as a two tailed alpha <0.1.

## Results

A total of 914 subjects, comprising 275 males and 639 females, were included in the study and completed relative clinical examinations. Of these, 301 participants (32.93%) were diagnosed with sleep disturbance. 639 and 275 participants were assigned to the training and validation sets, respectively, as a result of the random assignment in a 7:3 ratio. We compared each variable between patients with sleep disturbance and healthy controls in both groups, as detailed in Table 1.

**Table 1:** The demographic characteristics, hematological parameters, and EGEG variables of the middle-aged and elderly subjects in training and validation set.

Variables	Training set (*N* = 639)			Validation set (*N* = 275)		
	Sleep disturbance (*N* = 219)	Health control (*N* = 420)	*p* value	Sleep disturbance (*N* = 82)	Health control (*N* = 193)	*p* value
Demographic characteristics						
Gender			0.092^*^			0.289
Male	160 (73.06)	278 (66.19)		64 (78.05)	137 (70.98)	
Female	59 (26.94)	142 (33.81)		18 (21.95)	56 (29.02)	
Smoking	30 (13.70)	51 (12.14)	0.663	9 (10.98)	23 (11.92)	0.986
Alcohol	50 (22.83)	100 (23.81)	0.858	12 (14.63)	43 (22.28)	0.199
Age	57.39 ± 6.29	55.63 ± 5.92	0.001^***^	56.73 ± 6.14	55.11 ± 6.35	0.050^**^
BMI	24.11 ± 3.27	24.22 ± 2.89	0.654	24.07 ± 3.38	24.64 ± 3.26	0.195
Hematological parameters						
Glucose	5.59 ± 1.57	5.45 ± 1.19	0.275	5.33 ± 1.15	5.39 ± 1.05	0.695
TG	1.65 ± 1.25	1.60 ± 1.07	0.620	1.51 ± 0.80	1.74 ± 1.72	0.128
Tch	5.47 ± 0.93	5.36 ± 0.97	0.143	5.30 ± 1.04	5.44 ± 0.99	0.291
HDL	1.80 ± 0.48	1.76 ± 0.49	0.339	1.78 ± 0.49	1.76 ± 0.50	0.756
LDL	3.07 ± 0.75	2.98 ± 0.70	0.167	2.96 ± 0.79	3.03 ± 0.71	0.457
Gastric channel pre-meal						
MAW	174.88 ± 93.31	174.28 ± 87.87	0.938	195.28 ± 97.57	168.92 ± 87.10	0.036^**^
MFW	3.50 ± 0.31	3.47 ± 0.32	0.279	3.42 ± 0.29	3.45 ± 0.32	0.485
ERD, %	20.93 ± 3.54	20.96 ± 3.75	0.906	21.49 ± 4.78	20.97 ± 3.60	0.374
RAW	62.54 ± 33.04	62.95 ± 31.61	0.880	69.45 ± 35.68	60.38 ± 30.69	0.047^**^
ESR	0.87 ± 1.51	1.17 ± 1.94	0.031^**^	0.62 ± 1.13	1.17 ± 1.81	0.002^***^
DF	3.02 ± 0.24	2.98 ± 0.25	0.054^*^	2.95 ± 0.24	3.00 ± 0.26	0.097^*^
DPR, %	60.98 ± 6.38	61.08 ± 6.19	0.861	62.40 ± 6.63	61.24 ± 5.95	0.175
PNSW, %	58.38 ± 8.00	59.36 ± 8.47	0.148	60.19 ± 8.81	59.49 ± 7.73	0.532
CP, %	90.81 ± 7.07	90.04 ± 7.98	0.216	89.70 ± 8.06	90.93 ± 6.89	0.228
Intestinal channel pre-meal						
MAW	186.70 ± 115.78	191.62 ± 117.46	0.612	167.35 ± 98.65	183.66 ± 111.59	0.230
MFW	12.81 ± 2.30	12.98 ± 2.40	0.376	12.12 ± 2.02	12.83 ± 2.26	0.011^**^
ERD, %	23.98 ± 5.88	23.34 ± 5.78	0.196	25.11 ± 4.97	23.01 ± 5.59	0.002^***^
RAW	70.06 ± 41.83	72.18 ± 43.04	0.547	62.71 ± 36.76	68.60 ± 40.19	0.240
ESR	0.38 ± 0.77	0.45 ± 0.92	0.333	0.49 ± 1.07	0.41 ± 0.79	0.545
DF	11.57 ± 2.66	11.80 ± 2.86	0.312	10.91 ± 2.44	11.61 ± 2.73	0.035^**^
DPR, %	30.16 ± 6.00	30.98 ± 6.25	0.108	30.71 ± 6.60	31.22 ± 6.23	0.557
PNSW, %	51.84 ± 11.28	52.43 ± 13.17	0.553	54.84 ± 10.80	52.79 ± 13.17	0.179
CP, %	82.32 ± 17.60	82.97 ± 18.24	0.665	82.78 ± 18.99	83.47 ± 16.57	0.776
Gastric channel post-meal						
MAW	206.86 ± 93.50	211.29 ± 94.21	0.571	221.58 ± 98.08	204.96 ± 106.15	0.212
MFW	3.42 ± 0.33	3.42 ± 0.31	0.871	3.37 ± 0.27	3.42 ± 0.29	0.108
ERD, %	20.47 ± 4.04	21.10 ± 4.00	0.062^*^	20.19 ± 3.47	20.68 ± 3.90	0.299
RAW	73.44 ± 33.55	75.93 ± 33.44	0.373	79.07 ± 34.85	72.71 ± 36.26	0.173
ESR	1.31 ± 2.33	1.10 ± 2.13	0.252	1.17 ± 2.73	1.15 ± 2.05	0.937
DF	2.98 ± 0.27	2.97 ± 0.29	0.720	2.96 ± 0.26	2.96 ± 0.27	0.858
DPR, %	62.86 ± 6.07	61.5 ± 6.08	0.007^***^	63.44 ± 6.39	62.60 ± 6.18	0.313
PNSW, %	60.95 ± 8.35	59.67 ± 8.26	0.065^*^	61.63 ± 8.23	61.11 ± 8.50	0.637
CP, %	93.90 ± 5.85	92.58 ± 7.27	0.013^**^	92.40 ± 7.82	93.11 ± 5.95	0.465
Intestinal channel post-meal						
MAW	177.24 ± 96.21	187.78 ± 97.48	0.192	160.37 ± 76.60	176.75 ± 92.01	0.129
MFW	12.01 ± 1.89	12.13 ± 2.16	0.490	11.28 ± 1.77	12.16 ± 2.22	0.001^***^
ERD, %	25.73 ± 4.95	25.04 ± 5.23	0.102	25.94 ± 4.54	25.03 ± 4.77	0.134
RAW	66.12 ± 34.92	69.95 ± 35.32	0.191	58.96 ± 27.24	65.90 ± 33.23	0.073^*^
ESR	0.54 ± 1.02	0.57 ± 1.33	0.770	0.71 ± 1.18	0.56 ± 1.03	0.322
DF	10.46 ± 2.10	10.81 ± 2.51	0.059^*^	10.05 ± 1.96	10.85 ± 2.44	0.004^***^
DPR, %	28.96 ± 4.79	29.79 ± 5.03	0.042^**^	29.34 ± 4.94	29.77 ± 4.54	0.505
PNSW, %	53.60 ± 9.30	53.96 ± 10.44	0.649	54.30 ± 8.32	53.73 ± 10.66	0.635
CP, %	89.85 ± 10.33	91.71 ± 10.25	0.031^**^	91.26 ± 9.83	90.92 ± 10.42	0.799

TG, triglyceride; Tch, total cholesterol; HDL, high density lipoprotein; LDL, low density lipoprotein; MAW, mean amplitude of waveform; MFW, mean frequency of waveform; ERD, electrical rhythm disturbance; RAW, reaction area of waveform; ESR, electrical spreading rate; DF, dominant frequency; DPR, dominant power ratio; PNSW, percentage of normal slow wave; CP, coupling percent.

Classification variables are described in the form of frequency (%); Statistical significance, *p < 0.10; **p < 0.05; ***p < 0.01.

According to the results, age, ESR and DF on the pre-meal gastric channel, as well as DF on the post-meal intestinal channel were shown to be significantly different between patients with sleep disturbance and healthy control in both sets (*p* < 0.1). In addition, variables that had significant differences between sleep disturbance and healthy control in the training set alone included gender, ERD, DPR, PNSW and CP on the post-meal gastric channel, DPR, PNSW, and CP on the intestinal gastric channel; in the validation set alone included MAW and RAW on the pre-meal gastric channel, MFW, ERD and DF on the pre-meal intestinal channel, MFW and RAW on the post-meal intestinal channel.

**Figure 2:**
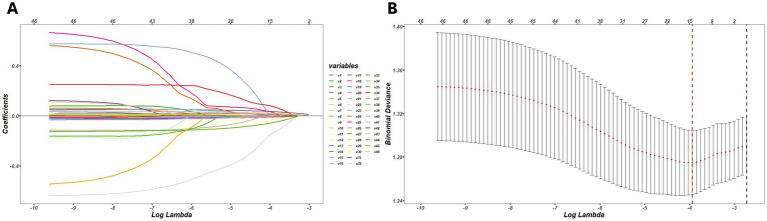
**(A)** The process of variables selection by binomial LASSO regression, a coefficient profile plot was produced against the log (lambda) sequence, different colors represent the different variables. **(B)** The results of ten-fold cross validation, by verifying the optimal lambda in the LASSO model, the partial likelihood deviance (binomial deviance) curve was plotted, and dotted vertical lines were drawn based on minimum lambda and standard error criteria.

Among the 46 associated variables, 13 potential predictors were chosen in the training set by binomial LASSO regression. These predictor variables were age, gender, LDL, ESR, DF, and PNSW on the pre-meal gastric channel, DPR on the pre-meal intestinal channel, RAW, DPR, and CP on the post-meal gastric channel, DF, DPR and CP on the post-meal intestinal channel. The variables screening processed by LASSO regression and ten-fold cross validation is shown in Figure 2, and the coefficients of retained variables are shown in Supplementary Sup S1.

**Figure 3:**
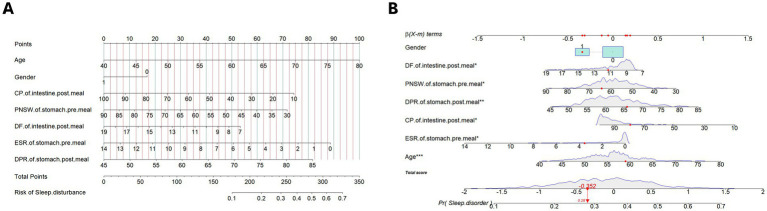
**(A)** The nomogram of the risk assessment model for sleep disturbance in training set. **(B)** The dynamic nomogram of the risk assessment model for sleep disturbance in training set. The nomogram of the sleep disturbance was developed with the predictors: age, gender, PNSW of gastric channel pre-meal, ESR of gastric channel pre-meal, DPR of gastric channel post-meal, CP of intestinal channel post-meal, DF of intestinal channel post-meal. PNSW, percentage of normal slow wave; ESR, electrical spreading rate; DPR, dominant power ratio; CP, coupling percent. *p < .05; **p < .01; ***p < .001.

The retained 13 predictors variables used stepwise binomial logistic regression for further optimization, and established the final risk assessment model. The final risk assessment model for sleep disturbance consists of seven predictors, including age, gender, PNSW and ESR on the pre-meal gastric channel, DPR on the post-meal gastric channel, DF and CP on the post-meal gastric channel. Among these predictors, increasing age associated with a higher risk of sleep disturbance, and male had a lower risk of sleep disturbance than female. With the exception of the DPR on the post-meal gastric channel, the EGEG variables were both negative associated with the risk of sleep disturbance, and details are presented in Table 2. All predictors except gender were significant at the 0.05 level. To facilitate risk assessment of sleep disturbance using these predictor variables, the nomogram and dynamic nomogram were both created and are shown in Figure 3, which is helpful to carry out personalized clinical evaluation.

**Table 2:** Risk assessment model of sleep disturbance in middle-aged and elderly people.

Variables	Beta	Std.error	Z value	OR	95%CI		*p* value
					Lower	Upper	
Age	0.050	0.015	3.446	1.052	1.022	1.082	0.001***
Gender, ref. = female	−0.340	0.193	−1.763	0.711	0.485	1.035	0.078*
*Pre-meal*							
PNSW of gastric channel	−0.024	0.011	−2.211	0.976	0.956	0.997	0.027**
ESR of gastric channel	−0.127	0.053	−2.381	0.881	0.790	0.974	0.017**
*Post-meal*							
DPR of gastric channel	0.041	0.015	2.774	1.042	1.012	1.073	0.006***
CP of intestinal channel	−0.017	0.008	−2.015	0.984	0.968	1.000	0.044**
DF of intestinal channel	−0.089	0.039	−2.283	0.914	0.845	0.986	0.022**

OR, odds ratio; ESR, electrical spreading rate; DF, dominant frequency; DPR, dominant power ratio; PNSW, percentage of normal slow wave; CP, coupling percent.

Classification variables are described in the form of frequency (%); Statistical significance, *p < 0.10; **p < 0.05; ***p < 0.01.

**Figure 4:**
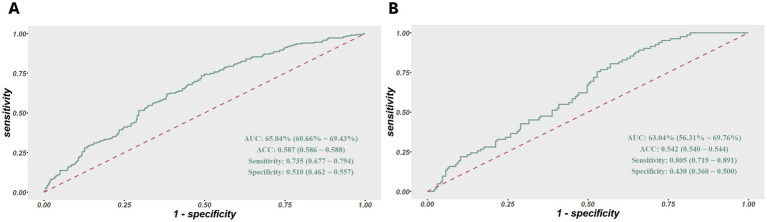
**(A)** ROC curves of the risk assessment model for sleep disturbance in the training set. **(B)** And in the validation set. The y-axis indicates the true-positive rate of the risk prediction. The x-axis indicates the false-positive rate of the risk prediction.

In terms of model validation and efficiency evaluation, we utilized the model to generate the predicted probability for 275 subjects in the validation set, and plotted the corresponding ROC curve to evaluate its sensitivity and specificity. The area under ROC curves were both above 0.6 in the two groups, indicating that the model exhibited a satisfying robustness. The results of sensitivity were 0.74 and 0.81 in the training and validation set respectively, meaning that the risk assessment model performed well in identifying true positive patients of sleep disturbance, but the results of specificity were 0.51 and 0.43, respectively, indicating that the model has insufficient ability to identify false negative patients, as shown in Figure 4.

**Figure 5:**
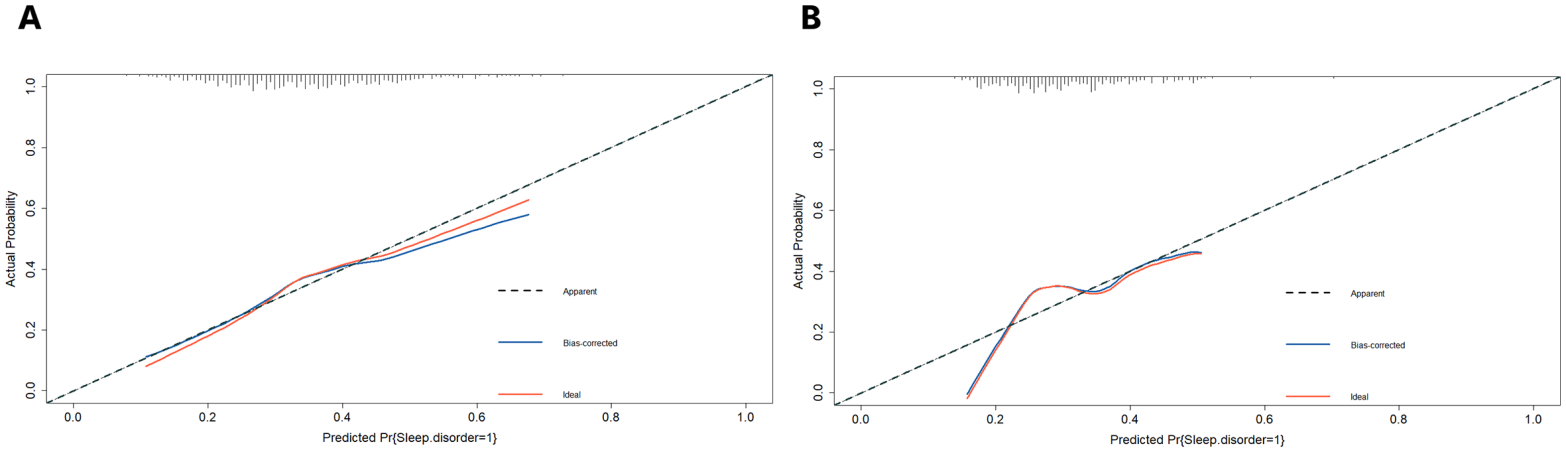
**(A)** The calibration curves of the risk assessment model for sleep disturbance in the training set. **(B)** And in the validation set. The y-axis indicates the actual probability of sleep disturbance. The x-axis indicates the predicted risk of sleep disturbance. The diagonal dotted line indicates perfect prediction by an ideal model. The solid line represents the model performance, a closer fit to the diagonal dotted line represents a better prediction.

**Figure 6:**
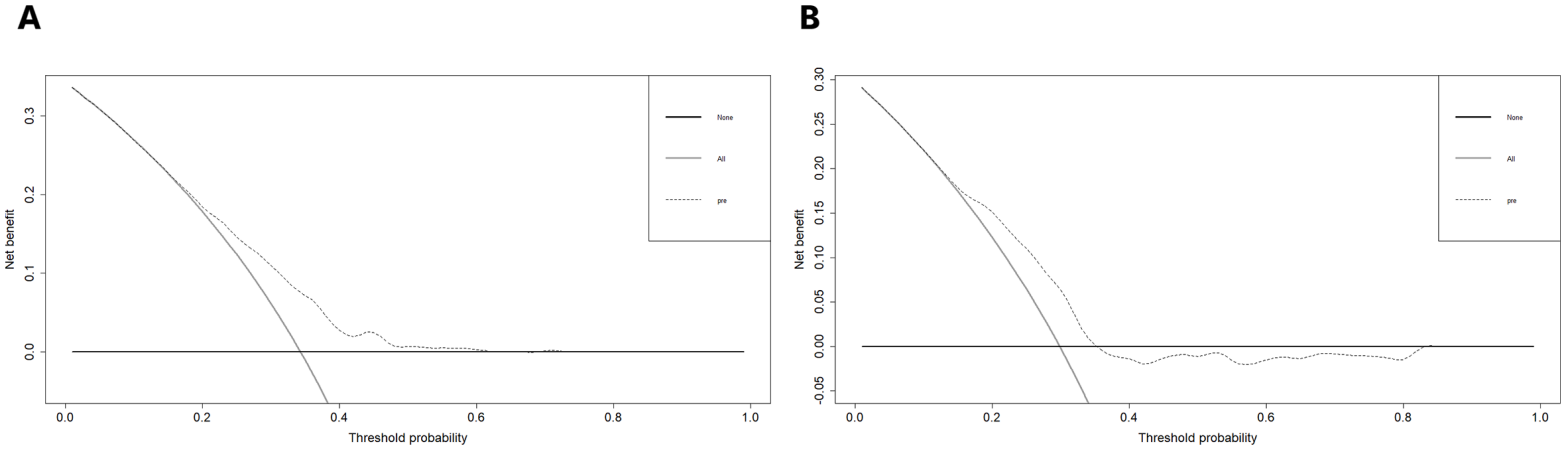
**(A)** Decision curve analysis of the risk assessment model for sleep disturbance in the training set. **(B)** And in the validation set. The y-axis measures the net benefit. The thick solid line represents the assumption that no patients have sleep disturbance. The thin solid line represents the assumption that all patients have sleep disturbance. The dotted line represents the risk nomogram.

Furthermore, we plotted the calibration curve and decision curve of the model both in two datasets, as shown in Figures 5, 6. The results of calibration curves in both datasets showed consistency between ideal curve and bias-correct curve; however, due to insufficient number of patients in the validation set, the curve deviated slightly from the apparent line. In the DCA, the threshold range of prediction probability were 0.34–0.63 and 0.29–0.35 in two sets, respectively, and the threshold probability selection range was significantly narrowed in the validation set. The overlap of threshold probabilities in the two sets is between 0.34 and 0.35. Therefore, in the EGEG-based risk assessment model, patients with a predicted probability higher than 0.35 were considered to be high risk of sleep disturbance, and this reference threshold might provide a clinical net benefit.

## Discussion

Sleep disturbance is a crucial factor affecting many nervous system diseases and mental disorders (33, 34). The development of sleep disturbance is also more likely to occur in middle-aged and elderly people. Improving the diagnosis rate of sleep disturbance in this population is conducive to earlier detection and treatment, which will significantly enhance their quality of life and reducing the burden of disease. However, extensive community screening is still difficult due to the limitations of self-reported questionnaires and polysomnography, leaving many people with sleep disturbance are never detected (35, 36). This deficiency is anticipated to be remedied by the risk assessment model of sleep disturbance based on EGEG that our research has developed. By using multiple evaluation indicators including ROC curve, calibration curve and DCA curve, the performance of the risk assessment model was compared and verified in the training set and verification set. It was discovered that the predictive ability of the model was stable and that it was effective at identifying positive patients with sleep disturbance. In the future, it is possible to use this model extensively in the community for sleep disturbance screening due to its satisfactory model performance.

For clinicians, the precise diagnostic of gastrointestinal inflammation, irritable bowel syndrome, functional gastrointestinal disorders, and digestive dysfunction is greatly benefited by the use of EGEG (37, 38). However, because the gastrointestinal channel has weaker myoelectrical activity than the heart and brain, there have not been many studies to explore EGEG’s potential for broader applications and connections to other disorders (39). Combining the risk prediction model developed based on EGEG for mild cognitive impairment (MCI) (40), EGEG demonstrated worthy predictive potential for both sleep disturbance and MCI. In the future, for middle-aged and elderly people with cognitive decline, communication disorders, and other difficulties to ensure the accuracy of questionnaire screening, EGEG may have excellent potential for detection of sleep disturbance and MCI.

In our risk assessment model, there are five parameters of EGEG contributed major value for predicting sleep disturbance, namely, PNSW and ESR on the pre-meal gastric channel, DPR on the post-meal gastric channel, DF and CP on the post-meal gastric channel. Comparing to the previous studies, PNSW, DF and CP also provide significant values in the diagnosis of gastrointestinal dysfunction (41, 42). For instance, a meta-analysis of EGG in patients with functional dyspepsia concluded that pre-meal PNSW and post-meal CP were important indicators (43). Another meta-analysis of EGG in patients with nausea and vomiting syndrome also found that post-meal DF was lower than that of healthy individuals (44). Our findings suggest that, in contrast to healthy individuals, the changes in gastrointestinal myoelectrical activity do exist not only in the sleep disturbance patients with gastrointestinal diseases, but also in the patients without the symptoms of gastrointestinal dysfunction.

Overall, our study demonstrates that sleep disturbance can be manifested by the changes of myoelectrical activity on the gastrointestinal channel. The risk assessment model established based on EGEG has important clinical significance and is promising to be used in the community screening for sleep disturbance in middle-aged and elderly people. However, our study still has some limitations. Since our subjects were recruited from the voluntary participation of middle-aged and elderly people in the community, and a perfect sampling procedure was not used, the representativeness of the samples is lacking, which cannot exclude potential selection bias. In addition, our study only included participants from the western region, which may have distinct population characteristics compared to other regions, and can be resolved by including other areas. In addition, the overall prediction accuracy of the model is less than 0.70, which may be due to the limited sample size, and may be clarified with a larger sample size.

## Conclusion

The risk assessment model based on EGEG indicators exhibits an acceptable efficiency and satisfying robustness of predicting the risk of sleep disturbance. Our findings also provide evidence for a close association between the gastrointestinal myoelectrical activity and sleep disturbance in middle-aged and elderly people. With the widely applied risk assessment model based on EGEG as an auxiliary method to diagnose sleep disturbance, it would be likely to achieve a full coverage of sleep disturbance screening for the middle-aged and elderly population in the community.

## Data availability statement

The original contributions presented in the study are included in the article/Supplementary materials, further inquiries can be directed to the corresponding author.

## Ethics statement

Written informed consent was obtained from the individual(s) for the publication of any potentially identifiable images or data included in this article.

## Author contributions

LC: conceptualization, methodology, investigation, resources, data acquisition, writing – review & editing, and supervision. SJ: methodology, formal analysis, writing-original draft, visualization, and writing – review & editing. BL: writing-original draft, methodology, and validation. CZ: data collection, formal analysis, data regulation, and supervision. YT: conceptualization and investigation. All authors contributed to the article and approved the submitted version.

## Funding

This study was supported by China National Key Research and Development Program (No. 2020AAA0105005) and 1-3-5 project for disciplines of Excellence Clinical Research Incubation Project, West China Hospital, Sichuan University (No. 2021HXFH012).

## Conflict of interest

The authors declare that the research was conducted in the absence of any commercial or financial relationships that could be construed as a potential conflict of interest.

## Publisher’s note

All claims expressed in this article are solely those of the authors and do not necessarily represent those of their affiliated organizations, or those of the publisher, the editors and the reviewers. Any product that may be evaluated in this article, or claim that may be made by its manufacturer, is not guaranteed or endorsed by the publisher.
