# Supplementary material for: Risk assessment model for sleep disturbance based on gastrointestinal myoelectrical activity in middle-aged and elderly people
Source: Front Psychiatry. 2023 Jun 23;14:1183108. doi: 10.3389/fpsyt.2023.1183108 (PMC10327604; doi:10.3389/fpsyt.2023.1183108)
Supplement: Supplementary file 1 [file Table_1.docx]

**Supplement**

**Sup.1 The retained variables were selected by binomial Lasso regression**

| Variables | Coefficients |
| --- | --- |
| Gender | -0.141487226 |
| Age | 0.03332254 |
| LDL | 0.027321153 |
| RAW post-meal gastric channel | -0.000196085 |
| DPR post-meal gastric channel | 0.019160598 |
| CP post-meal gastric channel | 0.009141213 |
| DF post-meal intestinal channel | -0.030299912 |
| DPR post-meal intestinal channel | -0.010223245 |
| CP post-meal intestinal channel | -0.006226472 |
| ESR pre-meal gastric channel | -0.055827289 |
| DF pre-meal gastric channel | 0.075695124 |
| PNSW pre-meal gastric channel | -0.006899919 |
| DPR pre-meal intestinal channel | -0.002181862 |
| Abbreviation: LDL, low density lipoprotein; RAW, reaction area of waveform; ESR, electrical spreading rate; DF, dominant frequency; DPR, dominant power ratio; PNSW, percentage of normal slow wave; CP, coupling percent. | |
